# Serum magnesium and the risk of prediabetes: a population-based cohort study

**DOI:** 10.1007/s00125-017-4224-4

**Published:** 2017-02-21

**Authors:** Brenda C. T. Kieboom, Symen Ligthart, Abbas Dehghan, Steef Kurstjens, Jeroen H. F. de Baaij, Oscar H. Franco, Albert Hofman, Robert Zietse, Bruno H. Stricker, Ewout J. Hoorn

**Affiliations:** 1 Department of Epidemiology, Erasmus University Medical Center Rotterdam, P.O. Box 2040, 3000 CA Rotterdam, the Netherlands; 2 Department of Internal Medicine, Erasmus University Medical Center Rotterdam, Rotterdam, the Netherlands; 3 Inspectorate for Health Care, Utrecht, the Netherlands; 4 Department of Physiology, Radboud Institute for Molecular Life Sciences, Radboud University Medical Center, Nijmegen, the Netherlands; 5 Department of Physiology, Anatomy and Genetics, University of Oxford, Oxford, UK; 6 Department of Epidemiology, Harvard T.H. Chan School of Public Health, Boston, MA USA

**Keywords:** Diabetes, Epidemiology, Insulin resistance, Magnesium, Magnesium regulating genes, Mediation, Population-based cohort, Prediabetes, Single nucleotide polymorphism

## Abstract

**Aims/hypothesis:**

Previous studies have found an association between serum magnesium and incident diabetes; however, this association may be due to reverse causation, whereby diabetes may induce urinary magnesium loss. In contrast, in prediabetes (defined as impaired fasting glucose), serum glucose levels are below the threshold for urinary magnesium wasting and, hence, unlikely to influence serum magnesium levels. Thus, to study the directionality of the association between serum magnesium levels and diabetes, we investigated its association with prediabetes. We also investigated whether magnesium-regulating genes influence diabetes risk through serum magnesium levels. Additionally, we quantified the effect of insulin resistance in the association between serum magnesium levels and diabetes risk.

**Methods:**

Within the population-based Rotterdam Study, we used Cox models, adjusted for age, sex, lifestyle factors, comorbidities, kidney function, serum levels of electrolytes and diuretic use, to study the association between serum magnesium and prediabetes/diabetes. In addition, we performed two mediation analyses: (1) to study if common genetic variation in eight magnesium-regulating genes influence diabetes risk through serum magnesium levels; and (2) to quantify the proportion of the effect of serum magnesium levels on diabetes that is mediated through insulin resistance (quantified by HOMA-IR).

**Results:**

A total of 8555 participants (mean age, 64.7 years; median follow-up, 5.7 years) with normal glucose levels (mean ± SD: 5.46 ± 0.58 mmol/l) at baseline were included. A 0.1 mmol/l decrease in serum magnesium level was associated with an increase in diabetes risk (HR 1.18 [95% CI 1.04, 1.33]), confirming findings from previous studies. Of interest, a similar association was found between serum magnesium levels and prediabetes risk (HR 1.12 [95% CI 1.01, 1.25]). Genetic variation in *CLDN19*, *CNNM2, FXYD2, SLC41A2,* and *TRPM6* significantly influenced diabetes risk (*p* < 0.05), and for *CNNM2, FXYD2, SLC41A2* and *TRPM6* this risk was completely mediated by serum magnesium levels. We found that 29.1% of the effect of serum magnesium levels on diabetes was mediated through insulin resistance, whereas for prediabetes 13.4% was mediated through insulin resistance.

**Conclusions/interpretation:**

Low serum magnesium levels are associated with an increased risk of prediabetes and this increased risk is similar to that of diabetes. Furthermore, common variants in magnesium-regulating genes modify diabetes risk through serum magnesium levels. Both findings support a potential causal role of magnesium in the development of diabetes, where the hypothesised pathway is partly mediated through insulin resistance.

**Electronic supplementary material:**

The online version of this article (doi:10.1007/s00125-017-4224-4) contains peer-reviewed but unedited supplementary material, which is available to authorised users.

## Introduction

The prevalence of diabetes mellitus is increasing worldwide and recent estimates indicate that one out of three people will develop diabetes during their life [1]. This underlines the importance of prevention and, therefore, the need to identify modifiable risk factors [1]. One potential modifiable risk factor that has emerged recently is magnesium. Magnesium is a co-factor in several pathways, including glucose transport, insulin sensitivity and insulin secretion [2–4], providing a molecular basis for its involvement in the pathogenesis of diabetes mellitus.

Several population-based cohort studies have identified associations between magnesium intake and incident diabetes, incident prediabetes (defined as impaired fasting glucose) and progression of prediabetes to diabetes [5–9]. However, the association between magnesium intake and diabetes may be explained by an overall healthier eating pattern, since healthy foods, such as green leafy vegetables, are usually magnesium rich. Therefore, magnesium intake could be a proxy for a healthy diet rather than an independent risk factor [10, 11]. These limitations may be partially addressed by using serum magnesium levels instead of magnesium intake as a determinant for association analyses. Indeed, lower serum magnesium levels have also been associated with diabetes [12, 13].

A remaining issue regarding the association between serum magnesium levels and diabetes is the possibility of reverse causality. Patients with diabetes show increased urinary magnesium loss, caused by hyperglycaemia, hyperfiltration or a direct effect of insulin on magnesium channels in the kidney [14]. In contrast, in prediabetes, serum glucose levels are below the threshold for urinary magnesium wasting and, hence, unlikely to influence serum magnesium levels.

In the current study, we aimed to explore the directionality of the relationship between serum magnesium levels and diabetes in a large population-based cohort with adjudicated endpoints and long-term follow-up. To do so, we studied the relationship between serum magnesium levels and prediabetes. An association between serum magnesium levels and this precursor stage of diabetes is not expected if low serum magnesium is caused by long-standing diabetes. To further address the directionality of the association between serum magnesium and diabetes, we investigated whether magnesium-regulating genes are associated with diabetes risk. Finally, we quantified the role of insulin resistance as a potential pathway by which serum magnesium levels influence diabetes risk.

## Methods

### Study design, setting and population

This study was embedded within the Rotterdam Study, a prospective population-based cohort study, ongoing since 1990 in a suburb of Rotterdam, the Netherlands. The rationale and design of this study have been described elsewhere [15]. In summary, the original cohort was extended with a second cohort in 2000 and a third cohort in 2006, resulting in a total study population of 14,926 participants, aged 45 years and older. Participants are asked to participate in follow-up examinations every 4–5 years. The Rotterdam Study complies with the Declaration of Helsinki and has been approved by the Medical Ethics Committee of the Erasmus Medical Center and by the Dutch Ministry of Health, Welfare and Sport, implementing the Wet Bevolkingsonderzoek: ERGO (Population Study Act: Rotterdam Study). All participants provided written informed consent to participate in the study and to obtain information from their physicians.

### Measurements

Magnesium was measured within blood collections from the third visit of the first cohort (1997–1999), and the baseline visits of the second (2000–2001) and the third (2006–2008) cohort. These visits are similar in design and methods for data collection. Magnesium (mmol/l) was measured in serum by the Department of Clinical Chemistry of the Erasmus Medical Center (Rotterdam, the Netherlands). All measurements were carried out using a colourimetric endpoint method and the Roche/Hitachi Cobas c501 Analyzer (Roche Diagnostics, Indianapolis, IN, USA). The CV for repeatability was 0.8% and the CV for intermediate precision was 1.4–1.7%.

Serum calcium, serum potassium, total cholesterol, HDL-cholesterol and serum creatinine were measured at the same visit as serum magnesium (Roche Diagnostics, Indianapolis, IN, USA). Serum calcium was measured using a photometric endpoint method, with a CV for repeatability of 0.4–2.0% and a CV for intermediate precision of 0.9–2.5%. Serum potassium was measured quantitatively with ion-selective electrodes, with a CV for repeatability of 0.3–0.6% and a CV for intermediate precision of 0.7–1.6%. eGFR was calculated using the Chronic Kidney Disease Epidemiology Collaboration (CKD-EPI) equation [16]. Total cholesterol and HDL-cholesterol were measured using an enzymatic colourimetric approach.

Measurement and assessment of anthropometrics has been described previously [15]. Briefly, blood pressure was measured twice during study visits and the average was calculated. Information on smoking habits and alcohol consumption was obtained during a home interview; smoking habits were categorised as non-smoker, former smoker and current smoker. Alcohol use was categorised into two categories: yes or no [15]. Information on prevalent stroke and coronary heart disease was determined on the date that blood was drawn from the participants via linkage with general practitioners working in the study area, and adjudicated by two medical doctors and a neurologist or cardiologist in case of disagreement [17]. Information regarding the use of diuretics was derived from linkage to pharmacy dispensing records [15]. Serum insulin was measured using an immunoassay (Roche Diagnostics) and for the majority of all participants (96.8%) fasting insulin levels were available. Insulin resistance was assessed using HOMA-IR values [18]. We used the natural logarithm (log_e_) of HOMA-IR levels +1 to account for non-normal distribution.

### Genetic analysis

We used a candidate gene approach to select 11 magnesium-regulating genes, which were hypothesized to play a role in the development of diabetes (see electronic supplementary material [ESM] Table 1). Lead single nucleotide polymorphisms (SNPs) in these magnesium regulating genes were identified from the literature [19–21]. Our goal was to select common genetic variations for our analysis; therefore, we only included SNPs with a minor allele frequency >10%, resulting in the following genes and corresponding SNPs being selected: *TRPM7* (rs8042919), *TRPM6* (rs2274924), *SLC41A1* (rs823154), *SLC41A2* (rs2463021), *CNNM2* (rs3740393), *CLDN19* (rs719676), *CLDN16* (rs9990270) and *FXYD2* (rs948100). Genotyping was carried out using the Illumina 550 duo and Illumina 610 quad BeadChip (Illumina, San Diego, CA, USA). For all participants that were included in this study, SNPs passed genotyping quality control, had a call rate >98% and had a Hardy–Weinberg *p* value >1 × 10^−6^. Data were imputed to the 1000 Genomes reference panel (phase 1, version 3) using MACH version 1.0.15/1.0.16 [22, 23]. Imputation quality for all SNPs was high (>0.97).

### Assessment of outcomes

In this study, we excluded prevalent cases of diabetes. Incident cases of prediabetes and type 2 diabetes were ascertained through active follow-up using general practitioners’ records, hospital discharge letters and glucose measurements from Rotterdam Study visits [1]. Diabetes and prediabetes were defined according to the recent WHO guidelines [24]. Briefly, diabetes was defined as fasting blood glucose ≥7.0 mmol/l, a non-fasting blood glucose ≥11.1 mmol/l (in the absence of fasting samples) or the use of blood glucose lowering medication (including oral blood glucose lowering medication such as metformin). Prediabetes was defined as a fasting blood glucose between 6.0 and 7.0 mmol/l or a non-fasting blood glucose between 7.7 and 11.1 mmol/l (in the absence of fasting samples). For the majority of all participants (96.8%) fasting blood glucose samples were available and for the remaining, a random non-fasting blood glucose level was used. Information regarding the use of blood glucose lowering medication was derived from both structured home interviews and linkage to pharmacy dispensing records [15]. All outcomes of prediabetes and diabetes were adjudicated by two independent study physicians. In case of disagreement, consensus was sought with an endocrinologist. Follow-up data was complete until 1 January 2012.

### Statistical analyses

Means, SD and percentages with frequency were used to report continuous and discrete variables. The relationship between serum magnesium levels and prediabetes was modelled using restricted cubic splines, using three knots, and was found to be linear. Therefore, we used serum magnesium as a continuous variable in multivariate Cox proportional hazards regression models to examine the relationship of serum magnesium levels with incident diabetes and prediabetes. Additionally, we studied the relationship between hypomagnesaemia (defined as serum magnesium ≤0.72 mmol/l) and incident diabetes and prediabetes. Follow-up time was calculated from the date that blood was drawn until the date of death, loss to follow-up or end of the study. The proportional hazards assumption was tested by plotting the log_10_ minus log_10_ survival curve and visually examining the curves, with no evidence that the assumption was violated [25].

We performed two mediation analyses: (1) quantifying the role of insulin resistance in the association of serum magnesium levels with diabetes and prediabetes; (2) to study if genetic variation in magnesium regulating genes associates with diabetes and prediabetes through serum magnesium levels. To do so, we used the logistic model as reported by Baron and Kenny [26]. We calculated the standardised indirect effect, which is a measure for the degree of mediation through the mediator, and tested for significance using bootstrapping procedures (*n* = 1000) [27]. For all analyses on diabetes we used a study population including all participants without diabetes at baseline; for the analyses on prediabetes we included all participants with normal glucose levels at baseline.

Missing data in covariables (present in 0.0–1.4%) were handled by single imputation using an expectation–maximization algorithm [28]. All analyses were repeated on complete cases to check for potential differences. With the exception of the baseline characteristics, results are reported for imputed data. We considered a two-sided *p* value <0.05 as statistically significant. Data were analysed using SPSS Statistics version 21.0 (IBM, Armonk, NY, USA) and R version 3.1.2. (The R Foundation for Statistical Computing, Vienna, Austria).

### Sensitivity analyses

To test the robustness of our findings, we performed several sensitivity analyses. First, we repeated our analysis on prediabetes using electrolytes other than magnesium (sodium, potassium, calcium and phosphate), to rule out that the observed association between serum magnesium levels and prediabetes is caused by a coexisting electrolyte disorder. In the second sensitivity analyses, we studied whether participants without a separate diagnosis date of prediabetes could have influenced our effect estimates. In these individuals, both the diagnosis of prediabetes and diabetes was regarded as being made on the same day, as no separate date of prediabetes diagnosis could be determined. Therefore, where the date of prediabetes diagnosis was missing, we imputed this date and repeated the analysis on prediabetes using these new dates. In the third sensitivity analysis, we excluded all participants with an eGFR below 60 ml min^−1^ [1.73 m^2^]^−1^ to exclude the possibility that impaired kidney function could have impacted our results. In the fourth sensitivity analysis, we excluded all participants with hypomagnesaemia and hypermagnesaemia to confirm that our results were not driven by these extremes. In the final sensitivity analysis, we studied if the association between serum magnesium and diabetes risk could have been explained by proton pump inhibitor use as proton pump inhibitors have been previously associated with hypomagnesaemia [29].

## Results

### Baseline characteristics

**Fig. 1:**
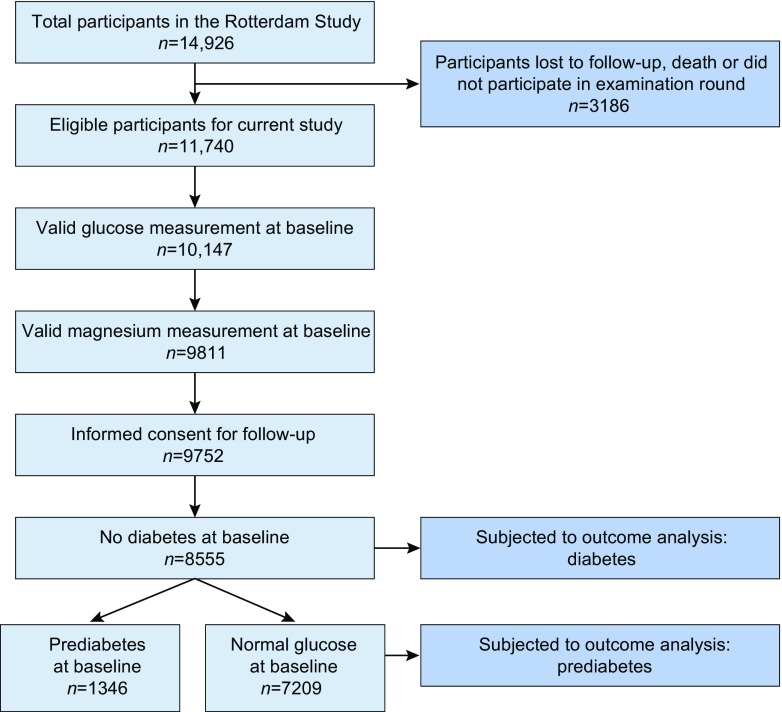
Flowchart of the study population. Of the 11,740 eligible participants, 3185 participants were excluded because of missing data in magnesium or glucose measurements at baseline, no informed consent or prevalent diabetes mellitus. This resulted in a total study population of 8555 participants, which could be further divided into 7209 participants with normal glucose level at baseline and 1346 participants with prediabetes at baseline

Our total study population comprised 8555 participants (Fig. 1). Table 1 shows the baseline characteristics of our study population. The mean age was 64.7 years and 57.8% were women. Participants with prevalent prediabetes more often had a history of smoking, hypertension, coronary heart disease, and used diuretics more often. The range of serum magnesium in the total population was 0.34–1.17 mmol/l. For participants with normal glucose levels at baseline, this range was 0.46–1.17 mmol/l, whilst for participants with prediabetes at baseline, this range was 0.34–1.20 mmol/l.

**Table 1:** Baseline characteristics of the study population

Characteristic	Total population *N* = 8555	Prevalent normoglycaemia *N* = 7209	Prevalent prediabetes *N* = 1346
Age, years	64.7 (9.7)	64.3 (9.7)	66.6 (9.4)
Women, *n* (%)	4949 (57.8)	4271 (59.2)	678 (50.4)
Body mass index, kg/m^2^	27.0 (4.0)	26.7 (3.9)	28.5 (4.4)
Smoking, *n* (valid per cent)			
Never	2647 (31.2)	2279 (31.9)	368 (27.4)
Former	3933 (46.3)	3272 (45.7)	661 (49.3)
Current	1914 (22.5)	1602 (22.4)	312 (23.3)
Alcohol use, *n* (valid per cent)	7297 (85.9)	6136 (85.8)	1161 (86.6)
Total cholesterol, mmol/l	5.76 (1.01)	5.76 (1.01)	5.74 (1.02)
HDL cholesterol, mmol/l	1.43 (0.41)	1.44 (0.41)	1.33 (0.40)
History of hypertension, *n* (valid per cent)	5078 (60.1)	4072 (57.2)	1006 (75.2)
History of stroke, *n* (valid per cent))	245 (2.9)	202 (2.8)	43 (3.2)
History of CHD, *n* (valid per cent)	516 (6.1)	409 (5.8)	107 (8.1)
eGFR (CKD-EPI), ml min^−1^ [1.73 m^2^]^−1^	79.7 (14.6)	80.0 (14.4)	78.3 (15.2)
Serum calcium, mmol/l	2.43 (0.10)	2.43 (0.10)	2.44 (0.10)
Serum potassium, mmol/l	4.35 (0.34)	4.36 (0.33)	4.34 (0.36)
Use of diuretics, *n* (%)	703 (8.2)	528 (7.3)	175 (13.0)
Serum glucose, mmol/l	5.46 (0.58)	5.30 (0.45)	6.32 (0.47)
Serum insulin, pmol/l	83.8 (63.0)	78.6 (56.0)	111.3 (84.8)
Serum magnesium, mmol/l	0.85 (0.06)	0.85 (0.06)	0.84 (0.06)
Hypomagnesaemia^a^, *n* (%)	131 (1.5)	92 (1.3)	39 (2.9)
Hypermagnesaemia, *n* (%)	185 (2.2)	155 (2.2)	30 (2.2)

Data are presented as *n* (%), *n* (valid per cent) or mean (SD)

Values are shown for non-imputed data

*n* values for certain variables do not necessarily match the total cohort number for each category because of missing data as a result of: (1) no answer provided during interview (smoking status and alcohol use); (2) unavailable blood pressure measurements during the examination round (history of hypertension); or (3) inability to link the study database to the General Practitioners’ database or registries and, therefore, not being able to ascertain disease status (history of stroke and coronary heart disease). For variables with missing data, valid per cent is given

^a^Serum magnesium ≤0.72 mmol/l

^b^Serum magnesium ≥0.97 mmol/l

### Association between serum magnesium levels and incident diabetes

Among the 8555 participants without diabetes at baseline, 806 cases of incident diabetes were identified over a median follow-up of 6.7 years. Table 2 shows the association between serum magnesium levels and incident diabetes. We found that a 0.1 mmol/l decrease in serum magnesium levels was associated with an increase in diabetes risk (HR 1.21 [95% CI 1.07, 1.37]). After adjustment for lifestyle factors, comorbidities and other electrolytes this association persisted, but was slightly attenuated (HR 1.18 [95% CI 1.04, 1.33]). Participants with hypomagnesaemia had an increased diabetes risk before and after adjustment for confounders (HR 2.12 [95% CI 1.38, 3.28] and HR 1.79 [95% CI 1.16, 2.77], respectively). We found no evidence of effect modification by sex (*p* for interaction = 0.36).

**Table 2:** Association between serum magnesium levels and incident diabetes and prediabetes

	At risk	Cases	Follow-up	HR (95% CI)		
Variable	(*N*)	(*N*)	(person-years)	Model 1	Model 2	Model 3
Diabetes						
Per 0.1 mmol/l decrease	8555	806	67,296	1.21 (1.07, 1.37)	1.17 (1.04, 1.32)	1.18 (1.04, 1.33)
No hypomagnesaemia	8424	785	66,421	1.00 (reference)	1.00 (reference)	1.00 (reference)
Hypomagnesaemia	131	21	875	2.12 (1.38, 3.28)	1.80 (1.17, 2.78)	1.79 (1.16, 2.77)
Prediabetes						
Per 0.1 mmol/l decrease	7209	1120	54,243	1.14 (1.02, 1.27)	1.13 (1.01, 1.25)	1.12 (1.01, 1.25)
No hypomagnesaemia	7117	1101	53,667	1.00 (reference)	1.00 (reference)	1.00 (reference)
Hypomagnesaemia	92	19	576	1.72 (1.09, 2.71)	1.51 (0.96, 2.37)	1.44 (0.91, 2.27)

Model 1: adjusted for age, age^2^, sex

Model 2: model 1+BMI, smoking status, alcohol use and total cholesterol:HDL-cholesterol ratio, history of hypertension, history of stroke and history of coronary heart disease

Model 3: model 2 + eGFR (CKD-EPI), serum calcium, serum potassium and use of diuretics

### Association between serum magnesium levels and incident prediabetes

Among the 7209 participants with blood glucose levels within the normal range (fasting blood glucose <6.0 mmol/l or non-fasting blood glucose <7.7 mmol/l) at baseline, 1120 cases of incident prediabetes were identified over a median follow-up of 5.7 years. Table 2 shows the association between serum magnesium levels and incident prediabetes. We found that a 0.1 mmol/l decrease in serum magnesium levels was associated with an increased risk of prediabetes before adjustment (HR 1.14 [95% CI 1.02, 1.27]) and after adjustment (HR 1.12 [95% CI 1.01, 1.25]) for confounders. Participants with hypomagnesaemia had an increased diabetes risk before adjustment for confounders (HR 1.72 [95% CI 1.09, 2.71]). After adjustment for confounders, this risk was attenuated and was no longer statistically significant (HR 1.44 [95% CI 0.91, 2.27]). We found no evidence of effect modification by sex (*p* for interaction = 0.18).

### Genetic risk factors

The selected common genetic variants were mainly located in introns (ESM Table 1). For five SNPs (rs719676, rs3740393, rs948100, rs2463021 and rs2274924), we found a significant association with serum magnesium levels (Table 3). For four SNPs (rs3740393, rs948100, rs2463021 and rs2274924), we found an indirect effect on diabetes risk. For rs719676, we found a significant direct effect on diabetes risk (OR 0.84 [95% CI 0.74, 0.96]), but no evidence that this effect was mediated through serum magnesium levels. The same analysis was repeated on prediabetes risk, but only a significant direct effect of rs719676 on prediabetes was observed (OR 0.88 [95% CI 0.78, 0.98]). The other selected gene variants were not significantly associated with prediabetes.

**Table 3:** Mediation analysis for magnesium-regulating genes

			Diabetes *N* = 7428		Prediabetes *N* = 6267	
Gene	SNP	Effect on serum magnesium levels^a^ β, mmol/l (95% CI)	Direct effect^b^ OR (95% CI)	Indirect effect^c^ OR (95% CI)	Direct effect^d^ OR (95% CI)	Indirect effect^e^ OR (95% CI)
*CLDN16*	rs9990270	0.001 (−0.001, 0.002)	0.92 (0.83, 1.03)	1.00 (1.00, 1.00)	0.99 (0.90, 1.09)	1.00 (1.00, 1.00)
*CLDN19*	rs719676	0.002 (0.000, 0.005)*	0.84 (0.74, 0.96)*	1.00 (0.99, 1.00)	0.88 (0.78, 0.98)*	1.00 (0.99, 1.00)
*CNNM2*	rs3740393	0.005 (0.003, 0.008)*	0.97 (0.82, 1.15)	0.99 (0.98, 1.00)*	1.07 (0.94, 1.23)	1.00 (0.99, 1.00)
*FXYD2*	rs948100	−0.004 (−0.006, −0.001)*	1.12 (0.94, 1.33)	1.01 (1.00, 1.02)*	1.14 (0.98, 1.32)	1.00 (1.00, 1.01)
*SLC41A1*	rs823154	0.000 (−0.001, 0.002)	0.94 (0.84, 1.05)	1.00 (1.00, 1.00)	0.95 (0.86, 1.04)	1.00 (1.00, 1.00)
*SLC41A2*	rs2463021	−0.004 (−0.007, 0.000)*	1.00 (0.82, 1.22)	1.01 (1.00, 1.02)*	0.96 (0.80, 1.14)	1.00 (1.00, 1.01)
*TRPM6*	rs2274924	−0.004 (−0.006, −0.001)*	1.01 (0.87, 1.16)	1.01 (1.00, 1.02)*	1.07 (0.94, 1.21)	1.00 (1.00, 1.01)
*TRPM7*	rs8042919	0.002 (−0.001, 0.005)	0.95 (0.78, 1.15)	1.00 (0.99, 1.00)	1.05 (0.90, 1.23)	1.00 (0.99, 1.00)

^a^Effect of gene on serum magnesium level (mmol/l), per allele increase

^b^Direct effect (OR) of gene on diabetes, adjusted for serum magnesium levels

^c^Indirect effect (OR) of gene on diabetes, mediated by serum magnesium levels

^d^Direct effect (OR) of gene on prediabetes, adjusted for serum magnesium levels

^e^Indirect effect (OR) of gene on prediabetes, mediated by serum magnesium levels

* *p* < 0.05

### Association between serum magnesium levels and insulin resistance

**Fig. 2:**
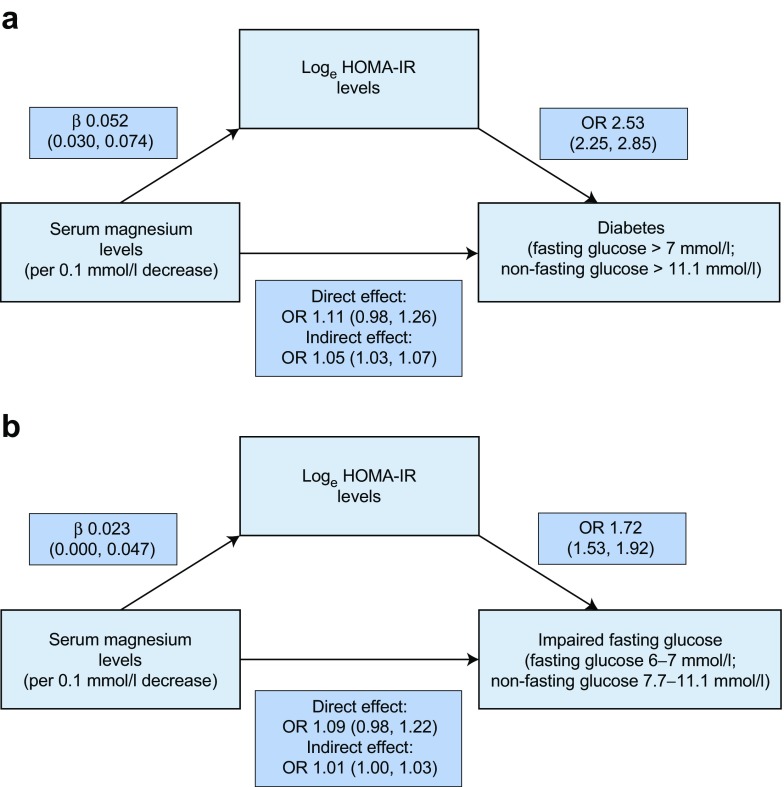
The role of insulin sensitivity in the association between serum magnesium levels and prediabetes/diabetes. The association was modelled using a mediation analysis, with insulin resistance calculated as log_e_ HOMA-IR levels. (**a**) The association with diabetes was studied in participants without diabetes at baseline. The direct effect of serum magnesium levels on incident diabetes was found not to be significant when adjusting for log_e_ HOMA-IR levels. The indirect effect, which represents the effect of serum magnesium levels on diabetes as mediated by log_e_ HOMA-IR levels, was found to be significant. (**b**) The association with prediabetes was studied in participants with normal blood glucose at baseline. The direct effect of serum magnesium levels on incident prediabetes was found not to be significant when adjusting for log_e_ HOMA-IR levels. The indirect effect was found to be significant

The association between serum magnesium levels, log_e_ HOMA-IR levels and diabetes is shown in Fig. 2a. We confirmed that serum magnesium levels were significantly associated with log_e_ HOMA-IR levels and also that log_e_ HOMA-IR levels were significantly associated with the risk of diabetes. When studying the direct effect of serum magnesium levels on the risk of diabetes adjusted for log_e_ HOMA-IR levels, the effect estimates were attenuated and the association was no longer statistically significant. The indirect effect of serum magnesium levels on diabetes risk, as mediated by log_e_ HOMA-IR levels, was significant with an odds ratio of 1.05 per 0.1 mmol/l decrease in serum magnesium levels. The calculated percentage of mediation was 29.1%. Fig. 2b shows the association between serum magnesium levels, log_e_ HOMA-IR levels and prediabetes. We found that serum magnesium levels were significantly associated with log_e_ HOMA-IR levels, within this group and that log_e_ HOMA-IR levels were also significantly associated with the risk of prediabetes, a finding similar to that of the association with diabetes. For the association with prediabetes, the calculated percentage of mediation was 13.4%.

### Sensitivity analyses

**Fig. 3:**
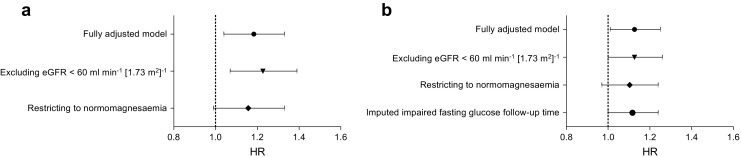
Sensitivity analyses. For both the analysis on (**a**) diabetes and (**b**) prediabetes we performed several sensitivity analyses, using the fully adjusted model (model 3). In the first analysis, we excluded all participants with an eGFR below 60 ml min^−1^ [1.73 m^2^]^−1^. In the second analysis, we excluded all participants with hypomagnesaemia or hypermagnesaemia. For the analysis on prediabetes we performed an additional sensitivity analyses to study if misclassified prediabetes cases (i.e. those without a prediabetes diagnosis date) could have influenced our results

We found that, besides magnesium, no other electrolyte was associated with the risk of prediabetes (Table 4). For 235 participants, we did not have a separate date for prediabetes diagnosis, therefore this date was imputed. For 25 participants, data were excluded from this sensitivity analysis as the imputed prediabetes diagnosis date was before serum magnesium measurement. The analysis on imputed prediabetes diagnosis date yielded similar results compared with that using the diabetes diagnosis date (Fig. 3b). In the subsequent two sensitivity analyses we excluded participants with an eGFR below 60 ml min^−1^ [1.73 m^2^]^−1^ and participants with hypomagnesaemia or hypermagnesaemia (Fig. 3). These analyses yielded similar results as in our main analyses. In the final analysis, we found that the addition of proton pump inhibitor use to our analysis on diabetes and prediabetes did not alter the association (data not shown).

**Table 4:** Sensitivity analysis using magnesium and other electrolytes as determinants

	Normal glucose to prediabetes	
Determinant	HR (95% CI)	*p* value
Serum magnesium (per 0.1 mmol/l decrease)	1.12 (1.01, 1.25)	0.034
Serum sodium (per 1 mmol/l decrease)	1.01 (0.99, 1.03)	0.297
Serum potassium (per 0.1 mmol/l decrease)	1.00 (0.98, 1.02)	0.821
Serum calcium (per 0.1 mmol/l decrease)	1.04 (0.98, 1.11)	0.218
Serum phosphate (per 0.1 mmol/l decrease)	1.03 (0.98, 1.07)	0.216

Model 3 (adjusted for age, age^2^, sex, BMI, smoking status, alcohol use, total cholesterol:HDL-cholesterol ratio, history of hypertension, history of stroke, history of coronary heart disease, eGFR (CKD-EPI), serum calcium, serum potassium and use of diuretics was used for all analyses

## Discussion

In this large population-based cohort, we found that over a median follow-up of almost 6 years, low serum magnesium levels are associated with an increased risk of prediabetes, with comparable risk estimates to that of diabetes. Furthermore, we found that common genetic variants in magnesium-regulating genes influence diabetes risk and that this risk is mediated through serum magnesium levels.

One strength of our study is that we thoroughly investigated the directionality of the association between serum magnesium and diabetes. Serum magnesium has already been associated with diabetes [12, 13]. However, increased renal magnesium wasting as a result of uncontrolled diabetes could lead to low serum magnesium levels [30]. This reversed causation may explain the observed association between serum magnesium and diabetes. If indeed low serum magnesium levels are only the result of uncontrolled diabetes, rather than a potential cause, one would not expect to find an effect of magnesium supplementation on diabetes risk and there would be no need to design a randomised controlled trial. Our study provides more evidence that magnesium may indeed influence diabetes risk, as we found similar associations for prediabetes as diabetes. The association with prediabetes is unlikely to be caused by reversed causation as glucose levels are not high enough to cause increased urinary magnesium wasting. Additionally, we found that magnesium-regulating genes associate with diabetes risk, further demonstrating that reverse causation is not likely to play a role, since diabetes status cannot alter genetic makeup. Another strength of our study is the detailed classification of diabetes and prediabetes, using a combination of prospectively gathered data that included the medical records of hospitals and general practitioners, electronic linkage with pharmacy dispensing records in the study area and standardised blood glucose measurements during visits to the study centre. This comprehensive assessment reduces potential bias resulting from misclassification. Furthermore, our study used a mediation analysis; this type of analysis goes beyond traditional analyses as it allows for a more causal interpretation of data, when certain assumptions are met [31]. The limitation of this type of analysis is directly linked to these assumptions, one of which is that there is no unmeasured confounding in the relationship between the mediator and the outcome. This assumption is difficult to check as unmeasured confounding can always play a role in observational data. However, we have adjusted our analysis for many potential confounders, which only slightly attenuated the effect estimates. Therefore, it is unlikely that the association between serum magnesium and diabetes risk is due to residual confounding, as this unknown confounder must be stronger and more imbalanced than all the confounders used in the current study. A second limitation of our study is that participants of the Rotterdam Study are predominantly of European descent. Previous studies demonstrated a lack of association between serum magnesium levels and diabetes risk in black participants [12], but we were unable to stratify on ethnicity. Another limitation of our study is the use of a single measurement of serum magnesium. Consequently, we were unable to study if changes in serum magnesium levels over time might influence diabetes risk. However, in a previous study serum magnesium concentrations measured 1 year apart were found to strongly correlate [32].

In combination with association directionality, we also studied the pathway by which serum magnesium levels may influence prediabetes and diabetes risk and found evidence of a dominant role of insulin resistance. A previous study demonstrated an effect of serum magnesium levels on insulin resistance [2]. However, the mediating effect of serum magnesium levels on diabetes through insulin resistance was not quantified. We found that approximately 29% of the effect of serum magnesium levels on diabetes and approximately 13% of the effect on prediabetes is mediated through insulin resistance. This partial mediation confirms the results of the Atherosclerosis Risk in Communities (ARIC) study, which found that the effect of serum magnesium levels on diabetes risk remained after adjusting for fasting glucose levels [12]. The remaining effect of serum magnesium levels on diabetes and prediabetes risk could occur via insulin secretion or through its impact on insulin signalling [33]. The effect of magnesium on insulin metabolism and glycaemic control, in both patients with diabetes and without diabetes, has been studied in several randomised clinical trials. A meta-analysis of 370 patients with type 2 diabetes found that oral magnesium supplementation of 360 mg/day could influence serum magnesium levels, although not linearly, after a median of 12 weeks. This oral magnesium supplementation also significantly lowered fasting glucose levels, but did not influence long-term glycaemic control [34]. In another trial, the effect of 16 weeks of 2.5 g magnesium chloride administration daily was studied in type 2 diabetes patients with decreased serum magnesium levels at baseline. Magnesium supplementation significantly increased serum magnesium concentration and significantly reduced fasting glucose levels, fasting insulin levels and HbA_1c_ levels compared with the control group [35]. Furthermore, in obese patients without hypomagnesaemia but with decreased insulin sensitivity at baseline, a positive effect of supplementation with 365 mg magnesium per day was observed. However, this supplementation only increased ionised magnesium levels and not total serum magnesium levels [36]. The effect of magnesium supplementation on insulin sensitivity was less pronounced in patients with normomagnesaemia. However, a positive effect could still be observed in these patients, supporting our finding of a linear association between serum magnesium levels and prediabetes risk.

We found that five previously identified magnesium-regulating genes were significantly associated with serum magnesium levels and that these genes were also associated with diabetes risk. We, hereby, replicate the findings from previous studies on the effect of *TRPM6, SLC41A2* and *CLDN19* on diabetes risk [20, 21]. The effect of genetic variation in *TRPM6* and *SLC41A2* was found to be mediated through serum magnesium levels. Variation in *TRPM6* can alter serum magnesium levels, as it has an essential role in magnesium reabsorption within the distal convoluted tubule [37] and *SLC41A2* is important for mediated magnesium transport across the plasma membrane [38]. *CLDN19* was found to associate with diabetes risk independently from serum magnesium levels. *CLDN19* is highly expressed in renal tubules where it influences paracellular magnesium transport. However, *CLDN19* has also been found in extra-renal tissues, including peripheral neurons where it was found to influence the pore selectivity of tight junctions [39]. Therefore, a magnesium-independent role for *CLDN19* in diabetes risk could be proposed since alterations in tight junctions have previously been linked with diabetes [40]. Besides confirming the role of these three genes in the association between magnesium and diabetes, we also identified new associations between two other magnesium-regulating genes (*CNNM2* and *FXYD2*) and diabetes risk, which were also mediated through serum magnesium levels. *CNNM2* is thought to play a role in intracellular magnesium sensing. However, it is still unclear how this may lead to alterations in magnesium transport and, thus, alterations in serum magnesium levels [37]. *FXYD2* encodes the γ-subunit of the Na^+^–K^+^-ATPase on the basolateral membrane of the distal convoluted tubule, where it drives paracellular magnesium transport through maintenance of the membrane potential [41]. At present, these findings have little impact on clinical or population health but they do provide more grounds for the role of magnesium in the pathogenesis of diabetes since slight alterations in serum magnesium levels, caused by common genetic variation in magnesium-handling genes, were found to impact diabetes risk. As expected with common genetic variants, the observed ORs were small. As the effect of magnesium on prediabetes is less pronounced than its effect on diabetes, we also expected smaller effects of magnesium-regulating genes on prediabetes risk. A post hoc power calculation showed that we had little power (2.9–17.2%) to detect significant differences in the mediation analysis on prediabetes; therefore, it is not surprising that we did not find significant associations with prediabetes for all magnesium-regulating genes.

### Conclusions

In conclusion, we found that low serum magnesium levels are associated with an increased risk of prediabetes, with similar effect estimates as compared with diabetes. The effect of serum magnesium on prediabetes and diabetes risk is partly mediated through insulin resistance. Furthermore, common genetic variation in magnesium regulating genes *TRPM6, CLDN19, SLC41A2, CNNM2* and *FXYD2* significantly modify the risk of diabetes through serum magnesium levels. Both findings support a potential causal role of magnesium in the development of diabetes and warrant future randomised controlled trials to study the effect of long-term magnesium supplementation on diabetes risk.

## Electronic supplementary material

Below is the link to the electronic supplementary material.

ESM Table 1 (PDF 93 kb)

## Abbreviations

CKD-EPI: Chronic kidney disease epidemiology collaboration
SNP: Single nucleotide polymorphism
