# Supplementary material for: Serum magnesium and the risk of prediabetes: a population-based cohort study
Source: Diabetologia. 2017 Feb 21;60(5):843–53. doi: 10.1007/s00125-017-4224-4 (PMC6518103; doi:10.1007/s00125-017-4224-4)
Supplement: Supplementary file 1 — (PDF 93 kb) [file 125_2017_4224_MOESM1_ESM.pdf]

ESM Table 1 Minor allele frequency distribution among diabetes cases and controls, among selected Single Nucleotide Polymorphisms in magnesium regulating genes.

					Minor Allele Frequency	
Locus	SNP	Location	Functional consequence	Major/Minor Allele	Cases	Controls
CLDN16	9990270	Chromosome 3	Non-encoding region	G/C	0.43	0.45
CLDN19	719676	Chromosome 1	Intron	A/G	0.22	0.25
CNNM1	6584273	Chromosome 10	Intron	G/A	0.03	0.03
CNNM2	3740393	Chromosome 10	Intron	G/C	0.13	0.14
FXYD2	948100	Chromosome 11	Intron	C/G	0.10	0.11
KCNJ11	1800467	Chromosome 11	Intron	G/C	0.03	0.03
MRS2	7738943	Chromosome 6	Intron	G/C	0.07	0.06
SCL41A1	823154	Chromosome 1	Intron	C/T	0.39	0.41
SCL41A2	2463021	Chromosome 12	Non-encoding region	A/T	0.10	0.09
TRPM6	2274924	Chromosome 9	Missense	T/C	0.16	0.17
TRPM7	8042919	Chromosome 15	Missense	G/A	0.11	0.10

refSNP numbers, location, functional consequence and major allele was obtained from the Single Nucleotide Polymorphism Database (dbSNP).
